# IL-17A activates the PI3K/AKT/mTOR pathway to regulate bronchial fibroblast autophagy-mediated airway remodeling: evidence from conditional IL-17RA-deficient mice

**DOI:** 10.3389/fphys.2026.1873777

**Published:** 2026-07-08

**Authors:** Yuting Liu, Jian Zhou, Xuan An, Yanhui Gu, Lanying Zhang, Yue Ma, Qingpiao Tang, Shengyi Yu, Yao Ouyang

**Affiliations:** Department of Respiratory and Critical Care Medicine, Affiliated Hospital of Zunyi Medical University, Zunyi, China

**Keywords:** IL-17A, IL-17RA, airway remodeling, airway fibrosis, bronchial fibroblast, autophagy, PI3K/AKT/mTOR pathway, inflammatory factor

## Abstract

**Background:**

Airway remodeling (AR) is a key pathological feature of chronic lung diseases and is closely associated with disease progression. IL-17A has been implicated in airway inflammation and fibrosis, but its role in AR and the underlying mechanisms remain incompletely understood. This study investigated whether IL-17A regulates bronchial fibroblast autophagy and airway remodeling through the PI3K/AKT/mTOR pathway.

**Methods:**

An AR mouse model was established by intratracheal administration of an adenovirus expressing IL-1β. Lung histopathology and pulmonary function were evaluated in wild-type mice and conditional IL-17RA-deficient mice. Primary mouse bronchial fibroblasts were used to assess the effects of IL-17A on autophagy-related proteins, inflammatory and profibrotic mediator secretion, collagen production, and PI3K/AKT/mTOR pathway activation. The autophagy inhibitor 3-methyladenine (3MA) was used to further examine the relationship between autophagy and pathway activation.

**Results:**

Conditional IL-17RA deficiency attenuated airway inflammation, collagen deposition, and pulmonary function impairment in AR mice. In primary bronchial fibroblasts, IL-17A decreased LC3II/I expression, increased p62 expression, promoted collagen I/III production, altered inflammatory and profibrotic mediator secretion, and activated the PI3K/AKT/mTOR pathway. These effects were reduced in fibroblasts with lower IL-17RA expression. Moreover, IL-17A partially reversed the inhibitory effect of 3MA on PI3K/AKT/mTOR phosphorylation.

**Conclusion:**

IL-17A/IL-17RA signaling contributes to airway remodeling by suppressing bronchial fibroblast autophagy and promoting inflammatory and fibrotic responses, at least partly through PI3K/AKT/mTOR pathway activation. These findings suggest that IL-17A may represent a potential therapeutic target for AR.

## 1 Introduction

Respiratory disease is an important cause of mortality and disability worldwide (Meghji et al., 2021). Airway remodeling (AR) is the basic pathological manifestation of various lung diseases including chronic obstructive pulmonary disease (COPD) and asthma, but the specific mechanisms of its development remain unclear (Camoretti-Mercado and Lockey, 2021; Christopoulou et al., 2023). AR primarily manifests as chronic inflammation (Liu et al., 2021; Cheng et al., 2024), with bronchial fibroblasts causing extracellular matrix formation (Ge et al., 2019), increased deposition of collagens, particularly collagen types I and III, and fibronectin leading to fibrosis in the small airways (Di et al., 2021; Liu et al., 2021; Liu et al., 2021; Cheng et al., 2024).

Cigarette smoking is undoubtedly the main cause of COPD and contributes to AR in asthma. A large number of animal models of COPD, have been described, in a multiple species, with rodents the most widely employed. In addition to cigarette smoke, lipopolysaccharide, protease and pollutant-induced models, adenoviruses with high expression of IL-1β have been reported to induce airway inflammation and fibrosis (Yanagisawa et al., 2017).Autophagy is a lysosome-dependent subcellular degradation pathway for cellular structures, misfolded proteins, and pathogens, allowing the recycling of cellular material, reconstruction, regeneration, and repair, serving as an important defense and protective, homeostatic mechanism in the body (Fang et al., 2016; Yanagisawa et al., 2017; Deleyto-Seldas and Efeyan, 2021; Di et al., 2021; Liu et al., 2021). Autophagy, is a complex process, involving multiple signaling pathways, among which the PI3K/AKT/mTOR pathway is crucial. mTOR, a large highly conserved protein kinase, exists in two forms, mTORC1 and mTORC2, with mTORC1 being the main negative regulator of autophagy (Deleyto-Seldas and Efeyan, 2021). Dysregulated autophagy in cancer-associated fibroblasts and stromal fibroblasts is associated with tumor growth (Rudnick et al., 2021; Yuan et al., 2022). in addition, restoration of optimal levels of autophagy in lung fibroblasts can reduce collagen deposition and slow the progression of pulmonary fibrosis (Lee et al., 2024).

The interleukin-17 (IL-17) family comprises pro-inflammatory cytokines, among which IL-17A is mainly secreted by Th17 cells. IL-17 levels increase in areas of inflammation, leading to synergistic amplification of the inflammatory response with various other cytokines, and participating in the progression of multiple diseases (Huangfu et al., 2023). IL-17A is an important member of the IL-17 family. IL-17A binds to a heterodimer receptor complex composed of the IL-17RA and IL-17RC receptors, leading to signal transduction and various biological effects. Inhibition of IL-17RA has been shown to block the biological function of IL-17A (Lorè et al., 2016). Studies have demonstrated that IL-17A is involved in autophagy in various cells mediated by the PI3K/AKT/mTOR signaling pathway (Varshney and Saini, 2018; Zhou et al., 2020). IL-17A has been reported to promote AR by affecting small airway fibrosis (Yanagisawa et al., 2017), although the specific mechanisms remain unclear.

The aim of this study was to examine the effect of cytokine IL-17A on airway fibrosis and AR. We investigated adenovirus transfection of IL-1β (and control transfection) on airway inflammation, fibrosis and lung function in wild-type mice and in a specific IL-17RA knockout. Previous related studies have mainly explored the response of IL-17A inflammatory cells, without verifying the effect on lung function or further exploring the mechanism of action (Huang et al., 2025). Our previous study demonstrated that IL-17A contributes to COPD-related airway remodeling through activation of the PI3K/AKT/mTOR pathway and suppression of autophagy in mouse bronchial fibroblasts. However, that study mainly relied on pharmacological autophagy inhibition and lentivirus-mediated IL-17RA overexpression or silencing in cultured MBFs. In contrast, the present study was designed to extend these findings using an IL-17RA-deficient mouse model, thereby providing *in vivo* evidence supporting the involvement of the IL-17A/IL-17RA axis in airway remodeling. In addition, primary bronchial fibroblasts isolated from WT and IL-17RA-deficient mice were used to compare the effects of IL-17A, 3-MA, and their combination on autophagy, inflammatory cytokine production, collagen expression, and PI3K/AKT/mTOR pathway activation. Therefore, the present work differs from the previous study by focusing on IL-17RA deficiency *in vivo* and IL-17A-associated responses in primary bronchial fibroblasts with reduced IL-17RA expression, rather than only modulating IL-17RA expression *in vitro*.

## 2 Materials and methods

### 2.1 Mice feeding

Male C57BL/6 mice (6–8 weeks old, 18–22 g) were purchased from Hunan SJA Laboratory Animal Co., Ltd. Conditional IL-17RA-deficient mice, IL-17RA^flox/flox^; Sftpc-Cre^+^ mice on a C57BL/6 background (male, weight: 18–22 g, age: 6–8 weeks) were purchased from Cyagen Biotechnology Co., Ltd (Suzhou, China).

Mice used for the *in vivo* experiments and those used for primary bronchial fibroblast isolation were from separate cohorts. For the *in vivo* experiments, mice were divided into four groups: WT-Control, WT-AR, IL-17RA^-/--^Control, and IL-17RA^-/--^AR, with n = 5 mice per group. These mice were used for pathological assessment and pulmonary function analysis.

For primary bronchial fibroblast isolation, a separate cohort of WT-AR mice and conditional IL-17RA-deficient AR mice was used. For each genotype, five successful independent primary bronchial fibroblast preparations were included in the final experiments, and each preparation was generated by pooling bronchial tissues from five mice. Preparations that failed because of contamination or poor cell status were excluded before treatment and data analysis and were not counted as biological replicates.

Mice were housed in specific pathogen-free facilities at the Key Laboratory of Basic Pharmacology of Zunyi Medical University under controlled conditions, with a relative humidity of 55–70%, a temperature of 23–26 °C, free access to food and water, and a 12 h light/dark cycle. All animal experiments were conducted in accordance with the Guidelines for the Ethical Review of Laboratory Animal Welfare (GB/T 35892-2018)[https://std.samr.gov.cn/] (China SAo, 2018) and were approved by the Animal Experiments Committee of Zunyi Medical University (Ethical Review No. (2020) 2-007).

During the experiment, mice were observed once daily for general health indicators. After completing all experiments, all mice were sacrificed by quick cervical dislocation. Death was verified by monitoring the symptoms such as the absence of chest movement, lack of a detectable heartbeat, pale mucous membranes, no response to toe pinch and changes in eye color. Mouse body weight loss >20% was regarded as a humane endpoint in the present study. None of the experimental animals reached these criteria. The animal grouping and sample size for the in vivo and in vitro experiments are summarized in **Table 1**.

**Table 1:** Animal grouping and sample size for *in vivo* and *in vitro* experiments.

Experimental purpose	Group	Sample size used for final analysis
*In vivo* pathological and pulmonary function analysis	WT-Control	n = 5 mice
*In vivo* pathological and pulmonary function analysis	WT-AR	n = 5 mice
*In vivo* pathological and pulmonary function analysis	IL-17RA^-/--^Control	n = 5 mice
*In vivo* pathological and pulmonary function analysis	IL-17RA^-/--^AR	n = 5 mice
Primary bronchial fibroblast isolation	WT-AR	n = 5 independent preparations; 5 mice pooled per preparation
Primary bronchial fibroblast isolation	IL-17RA^-/--^AR	n = 5 independent preparations; 5 mice pooled per preparation

### 2.2 Adenovirus with high expression of IL-1β

Adenoviruses expressing high levels of IL-1β (Ad-IL-1β) with an initial titer of 4.0 x 10^11^ pfu/mL, as well as the control virus (Ad-LacZ) with an initial titer of 3.16 x 10^11^ pfu/mL, were purchased from Hanbio Biological Co., Ltd. Viruses were diluted to 75 μL in sterile PBS containing 2.5 x 10^8^ pfu in line with previous studies by Huang and Wu (Hashimoto et al., 2015; Yanagisawa et al., 2017).

### 2.3 Establishment of the animal model

Experiments were conducted in two main groups of animals; wild-type (WT) and IL-17RA-deficient (IL-17RA^-/-^) groups, each then subdivided into two subgroups, namely, the Control and AR subgroups. Following previous published methods (Hashimoto et al., 2015; Yanagisawa et al., 2017), mice were anesthetized with 1.25% avertin (250 mg/kg) via intraperitoneal injection. Adequate anesthetic depth was confirmed by the absence of the pedal withdrawal reflex before intratracheal procedures, followed by intratracheal injection of 75 μL Ad-IL-1β (the COPD group) or Ad-LacZ (the Control group). During animal experiments, mice were anesthetized with Avertin only once or at most twice, with at least one week between doses. No adverse effects such as respiratory depression, poor recovery, or behavioral abnormalities were observed during or after anesthesia. All animals recovered fully and behaved normally between procedures. Lung function was assessed on Day 8, after which mice were humanely euthanized by cervical dislocation under deep anesthesia in accordance with the American Veterinary Medical Association (AVMA) Guidelines for the Euthanasia of Animals (2020). All procedures were performed by trained personnel to minimize animal suffering, and tissues were subsequently collected for histopathological examination.

### 2.4 Histopathological examination

Mouse lung tissues were fixed in 4% paraformaldehyde for one week, dehydrated, and embedded in paraffin. The embedded tissues were sectioned (3-5 μm thickness) and stained with hematoxylin and eosin (HE) (Solarbio, China) or Masson’s stain (Solarbio, China) for evaluation of airway inflammation and collagen deposition, respectively. Inflammation was scored using a five-point scoring system (Ma et al., 2022) as follows: normal = 0; a few inflammatory cells = 1; one ring of inflammatory cells = 2; two to four rings of inflammatory cells = 3; > 4 rings of inflammatory cells = 4. Collagen deposition was assessed by determining the ratio of the area of collagen deposition (blue) to the total surface area (red) after Masson staining (Ma et al., 2022). Images of the stained sections were acquired using the Panoramic MIDI II 3DHISTECH digital pathology system and were analyzed using ImageJ (V1.8.0, NIH, USA) software.

### 2.5 Lung function tests

One week after modeling, mice were anesthetized by intraperitoneal injection of 1.25% avertin (250 mg/kg). Adequate anesthetic depth was confirmed by the absence of the pedal withdrawal reflex before the procedure. The parameters of the Animal Lung Function Measurement System (EMMS, UK) were set according to the manufacturer’s instructions. After anesthesia, the mice were fixed in the special support for the lung function instrument, the trachea was exposed in the same way as in the modeling, and a T-shaped incision was made in the trachea and a tracheal tube was inserted, which was connected to the corresponding tubes and connected to the lung function test instrument. Values of exertional expiratory volume, exertional lung volume and dynamic lung compliance were measured in mice during the first 0.1 s.

### 2.6 Cell culture

Primary bronchial fibroblasts were isolated from a separate cohort of WT-AR mice and conditional IL-17RA-deficient AR mice (IL-17RA^flox/flox^; Sftpc-Cre^+^). For each genotype, five independent primary fibroblast preparations were obtained, and each preparation was generated by pooling bronchial tissues from five mice.

Cells were cultured in high-glucose Dulbecco’s modified Eagle’s medium supplemented with 15% fetal bovine serum, penicillin-streptomycin, and glutamine at 37 °C in 5% CO_2_. Cells from passages 2–4 were used for subsequent experiments. Fibroblast identity was confirmed by immunofluorescence staining for vimentin, with DAPI used for nuclear staining.

The cells were divided into two main groups, WT and IL-17RA-/-, and each group was further subdivided into four treatment subgroups: Control, 3MA, IL-17A, and 3MA+IL-17A. The 3MA group was treated with 5 mM 3MA (MCE) for 24 h. The IL-17A group was treated with recombinant mouse IL-17A (PeproTech, Cat. No. 210-17) at 10 ng/mL for 24 h. This concentration was selected based on our previous study (Ding et al., 2025) and was further supported by preliminary CCK-8 assay results. The 3MA+IL-17A group was pretreated with 5 mM 3MA for 2 h before IL-17A stimulation for 24 h. The Control group received no treatment.

### 2.7 Immunofluorescence assay

Transfected cells were seeded into round coverslips in 24-well plates. After 24 h, the medium was removed and cells were washed by PBS. Then cells were fixed by 4% paraformaldehyde for 30 min and then washed 3 times with PBS. Cells were blocked with 5% bovine serum albumin (BSA) for 1 h. Cells on the round coverslip were incubated with Vimentin (1:100, #PB9359, Wuhan Doctor Bio-engineering Co.) primary antibodies overnight at 4 °C. After being washed by PBS for 3 times, secondary antibodies (1:200, #BA1127, Wuhan Doctor Bio-engineering Co.) were added to incubate for 2 h at room temperature. Finally, cells were stained with DAPI for nuclei, coverslips were inverted on slides, and fluorescence intensity was observed by fluorescence microscopy.

### 2.8 Transmission electron microscopy

Adherent MBFs were washed twice with pre-cooled PBS, and then collected and fixed with 2.5% glutaraldehyde at 4 °C for 24 h. The samples were then treated with 1% osmic acid fixative solution for 2 h, dehydrated through graded acetone, and further embedded in resin embedding agent. Ultrathin sections were prepared, stained with uranyl acetate and lead citrate, and examined by electron microscopy JEM-1400 FLASH (JEOL, Japan).

### 2.9 Enzyme-linked immunosorbent assay

ELISA kits were used to measure levels of IL-10 (#LJS-M-006, Lingjiesi Biotechnology), transforming growth factor beta (TGF-β) (#LJS-M-015, Lingjiesi Biotechnology), and CCL20 (#LJS-M-157, Lingjiesi Biotechnology) in the culture supernatants of bronchial fibroblasts. Sandwich ELISAs were performed, and optical density (OD) was measured at 450 nm using a microplate reader. The concentrations of mouse IL-10/TGF-β/CCL20 in the samples were calculated based on standard curves.

### 2.10 Western blotting

WB was performed to assess the expression of bronchial fibroblast proteins associated with signaling pathways, autophagy, and fibrosis. Treated bronchial fibroblasts were lysed with radioimmunoprecipitation assay (RIPA) buffer, and protein concentrations were determined using a bicinchoninic (BCA) protein assay kit. Proteins were separated on 7.5% or 12.5% sodium dodecyl sulfate-polyacrylamide (SDS) gels, transferred to nitrocellulose membranes (Bio-Rad), and blocked with protein-free rapid blocking solution (EpiZyme). The membranes were probed overnight at 4 °C with specific antibodies against components of signaling pathways: PI3K (1:1000, #4292, CST), p-PI3K (1:1000, #17366S, CST), AKT (1:1000, #9272, CST), p-AKT (1:1000, #4060T, CST), mTOR (1:1000, #2972S, CST), and p-mTOR (1:1000, #2971, CST). Specific antibodies against autophagy proteins P62 (1:1000, #5114, CST), LC3II/I (1:1000, #4108, CST), type III collagen (1:1000, #A0817, ABclonal), and type I collagen (1:1000, #A21059, ABclonal), IL-17RA (1:1000, #A5163, ABclonal), and GAPDH (1:10000, #AC001, ABclonal) was used as a loading control. The secondary antibodies used were HRP-conjugated Goat anti-Rabbit IgG (H+L) (1:5000, #AS014, ABclonal), incubated for 2 h at room temperature, and the bands were visualized using Meilunbio^®^ fg super sensitive ECL luminescence reagent (#MA0186). Protein bands were visualized using the ChemiDoc Touch Imaging System (Bio-Rad), and the integrated optical density (IOD) was analyzed using Image-Pro Plus 6.0 software (Media Cybernetics, Inc.).

### 2.11 Statistical analysis

Three independent experiments were performed for each treatment group unless otherwise stated. Statistical analyses were performed using SPSS version 29.0 (IBM Corp., Armonk, NY, USA). Data are presented as the mean ± standard deviation (SD). Comparisons between two groups were performed using an unpaired Student’s t-test. Comparisons among multiple groups involving one factor were performed using one-way ANOVA followed by Tukey’s *post hoc* test. For experiments involving two independent factors, such as genotype and treatment or genotype and model, two-way ANOVA followed by Tukey’s *post hoc* test was used. A value of P < 0.05 was considered statistically significant.

## 3 Results

### 3.1 IL-17A promoted airway inflammation and fibrosis in mice

**Figure 1:**
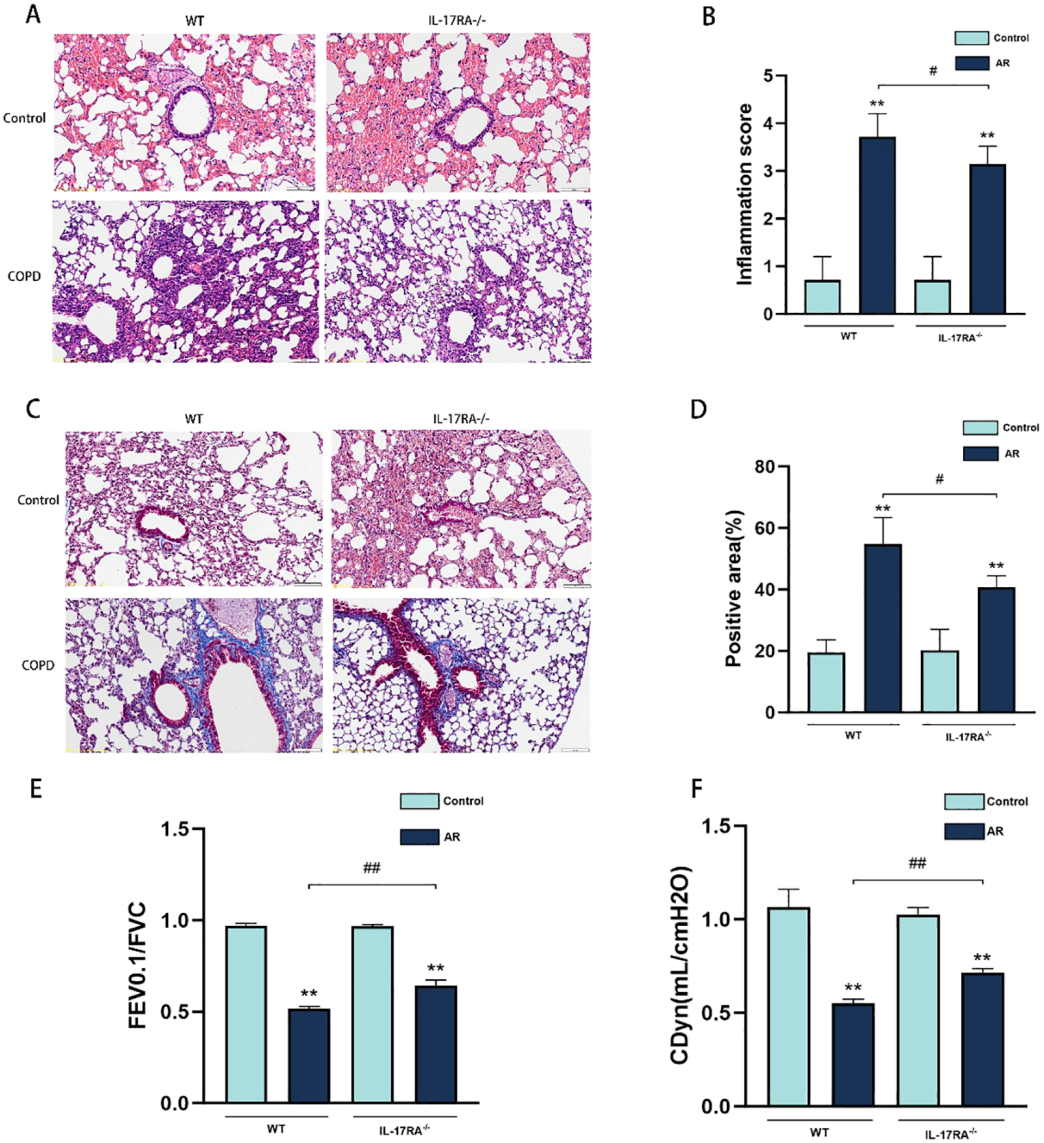
IL-17A promoted airway inflammation and fibrosis in mice. **(A)** Representative images of HE-stained lung tissue sections in each group (scale = 100 μm); **(B)** Inflammation scores were evaluated by HE staining; **(C)** Representative images of Masson-stained lung tissue sections in each group (scale = 100 μm); **(D)** Quantitative analysis of collagen deposition after Masson staining; E-F. Analysis of pulmonary function in mice in each group. Data are presented as mean ± SD. Statistical analysis was performed using two-way ANOVA followed by Tukey’s *post hoc* test. ^*^P < 0.05 and ^**^P < 0.01 versus the genotype-matched Control group; ^#^P < 0.05 and ^##^P < 0.01 between the groups connected by brackets; ns, not significant.

HE staining of lung tissue of AR mice in the WT and IL-17RA^-/-^ groups showed significantly greater inflammatory cell infiltration around the small airways (P < 0.01) with airway wall and smooth muscle thickening, compared with the control group. IL-17RA knockout significantly reduced airway inflammatory cell infiltration in the AR group (P < 0.05), suggesting that IL-17A may promote airway inflammation in mice (**Figures 1A, B**). Masson staining showed significantly greater deposition of collagen fibers around the airways in the AR group compared with the control group (P < 0.01), consistent with airway fibrosis IL-17RA knockout significantly reduced collagen deposition in mouse lung tissue (P < 0.05), suggesting that IL-17A may stimulate airway fibrosis in mice (**Figures 1C, D**). Following intratracheal injection of Ad-IL-1β into WT and IL-17RA^-/-^ mice, FEV0.1/FVC and Cdyn were significantly reduced in both groups (P < 0.01), (**Figures 1E, F**). FEV0.1/FVC and Cdyn were significantly less reduced in the IL-17RA^-/-^ group compared with WT group (P < 0.05), suggesting that IL-17A reduced pulmonary function in mice.

### 3.2 Culture and identification of bronchial fibroblasts

**Figure 2:**
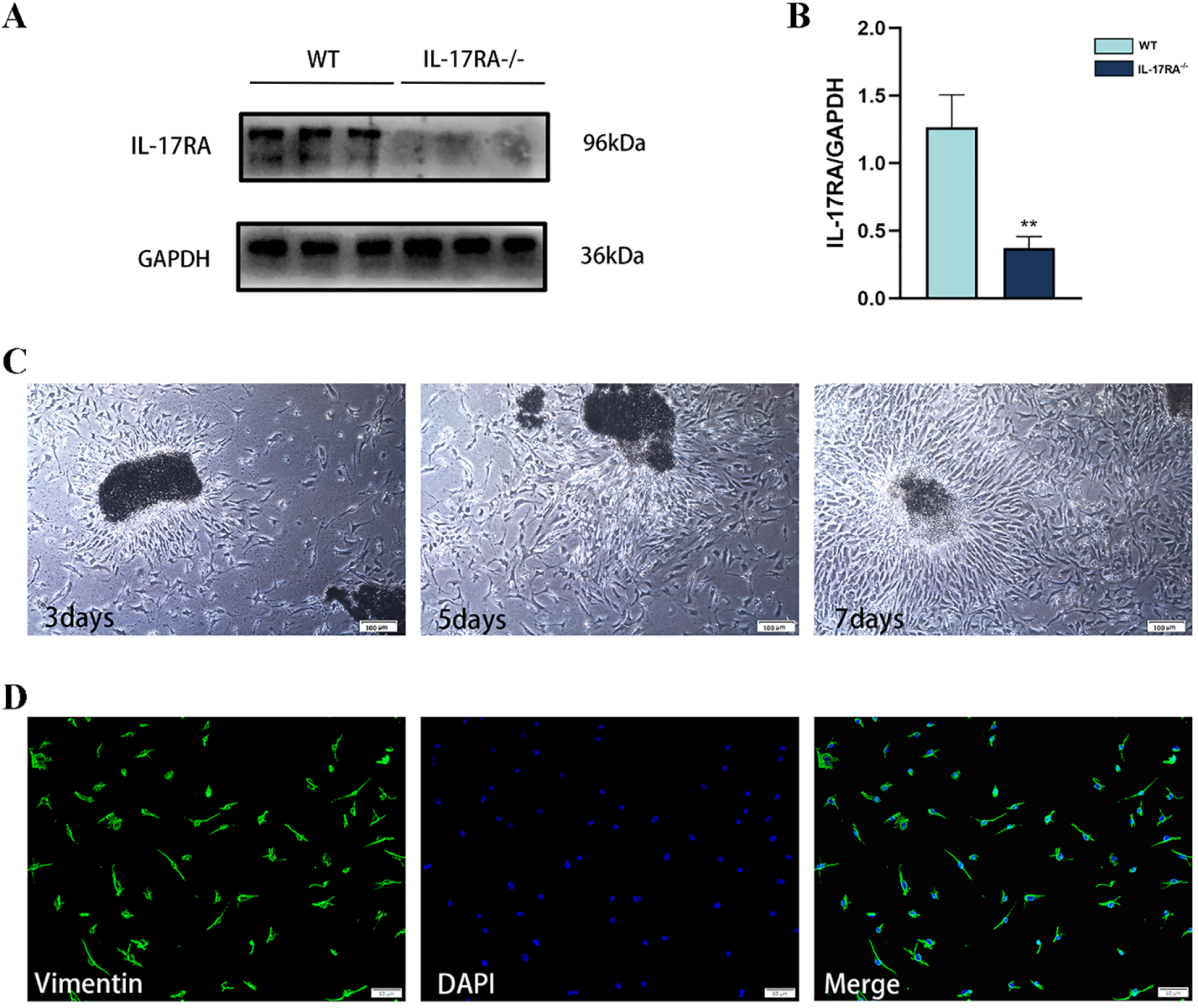
Culture of primary bronchial fibroblasts with immunofluorescence evaluation of passaged MBFs. A, **(B)** Representative protein bands on WB with quantitative determination of IL-17RA in the different groups (n=5); **(C)** Primary bronchial fibroblasts on Days 3, 5, and 7; **(D)** Demonstration of vimentin expression in passaged MBFs by immunofluorescence. DAPI staining for double-stranded DNA. Data are presented as mean ± SD. Statistical analysis was performed using an unpaired Student’s t-test. ^**^P < 0.01 versus WT mice.

The expression of IL-17RA in MBFs in each group was assessed using WB. The results showed (**Figures 2A, B**; n =5) that IL-17RA expression was significantly reduced in the IL-17RA^-/-^ group compared to the WT group (P < 0.01). After 3 days of adherent culture of bronchial tissue, individual mouse bronchial fibroblasts (MBFs) were observed to crawl out from the lung tissue block and adhere to the wall; these cells were characteristically triangular or spindle-shaped. After 5 days, the morphology of most MBFs was spindle-shaped, with a few triangular or polygonal cells, and they grew in clusters. After 7 days, cell clustering and growth were more pronounced, with cytoplasmic extensions and single-layer fusion, and the cells were arranged in parallel and showed radial growth (**Figure 2C**). Vimentin is an intermediate fibrillar protein present in fibroblasts and is commonly used as a surface marker to identify fibroblasts (Terriac et al., 2017; Ge et al., 2019). Therefore, the expression of the specific marker protein vimentin in passaged MBFs was identified by immunofluorescence (**Figure 2D**).

### 3.3 IL-17A inhibited autophagy in bronchial fibroblasts

**Figure 3:**
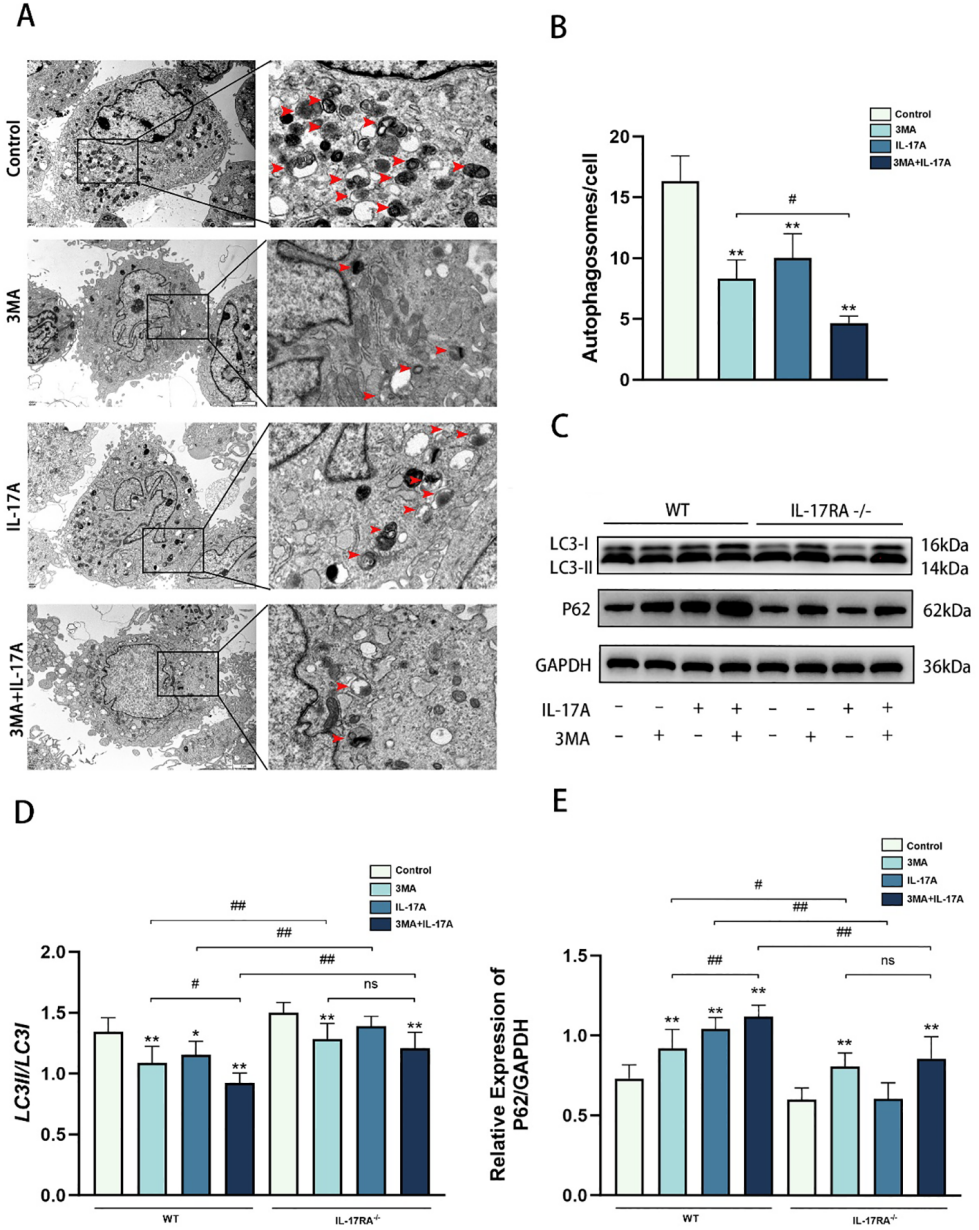
IL-17A inhibited autophagy in bronchial fibroblasts (x̄ ± SD, n=5). **(A)** Representative images of autophagosomes **(red arrow)** in MBF cells from each group. Left Scale = 2 μm, Right Scale = 500 nm, n = 5; **(B)** Quantitative determination of autophagosomes in MBF cells; **(C)** Representative protein bands in the different groups; **(D)** Quantitative determination of LC3II/LC3I in the different groups; **(E)** Quantitative determination of p62 in the different groups. Data are presented as mean ± SD. For **Figure 3B**, statistical analysis was performed using one-way ANOVA followed by Tukey’s *post hoc* test. For **Figures 3D, E**, statistical analysis was performed using two-way ANOVA followed by Tukey’s *post hoc* test. ^*^P < 0.05 and ^**^P < 0.01 versus the genotype-matched Control group; ^#^P < 0.05 and ^##^P < 0.01 between the groups connected by brackets; ns, not significant.

TEM was used to evaluate autophagy in MBFs from WT mice in the Control, 3MA, IL-17A, and 3MA+IL-17A groups (**Figures 3A, B**). Compared to the Control group, the 3MA, IL-17A, and 3MA+IL-17A groups exhibited reduced numbers of autophagosomes (P < 0.01). The 3MA+IL-17A group showed fewer autophagosomes compared to the 3MA group (P < 0.05). This suggests that IL-17A may inhibit the formation of autophagosomes in MBFs. The specific effects of IL-17A on MBF autophagy were examined in MBFs from WT and *IL-17RA^-/-^* mice divided into Control, 3MA, IL-17A, and 3MA+IL-17A groups. The expression of the autophagy proteins LC3II/I and p62 was evaluated in each group of MBFs using WB (**Figures 3C–E**). The following results were obtained: 1) In WT cells the 3MA, IL-17A, and 3MA+IL-17A groups all showed significantly reduced expression of LC3II/I (P < 0.05 or P < 0.01) and significantly increased expression of p62 (P < 0.01) compared with the Control group. LC3II/I expression was significantly reduced in the 3MA+IL-17A groups (P < 0.05) while p62 expression was significantly increased (P < 0.05) compared with the 3MA group. 2) In IL-17RA^-/-^ cells, the 3MA group and 3MA+IL-17A group showed significantly reduced LC3II/I expression (P < 0.01) and significantly increased p62 expression (P < 0.01), while the IL-17A group showed no significant difference compared with the Control group (P ≥ 0.05)., The 3MA+IL-17A group showed no significant change in the expression of LC3II/I and p62 compared with the 3MA group (P ≥ 0.05). 3), IL-17RA^-/-^ cells showed increased LC3II/I expression (P < 0.01) and decreased p62 expression (P < 0.05 or P < 0.01) in the 3MA, IL-17A, and 3MA+IL-17A groups compared with WT cells. This suggests that IL-17A inhibited the expression of autophagy-related proteins in MBFs.

### 3.4 IL-17A promoted collagen secretion by bronchial fibroblasts

**Figure 4:**
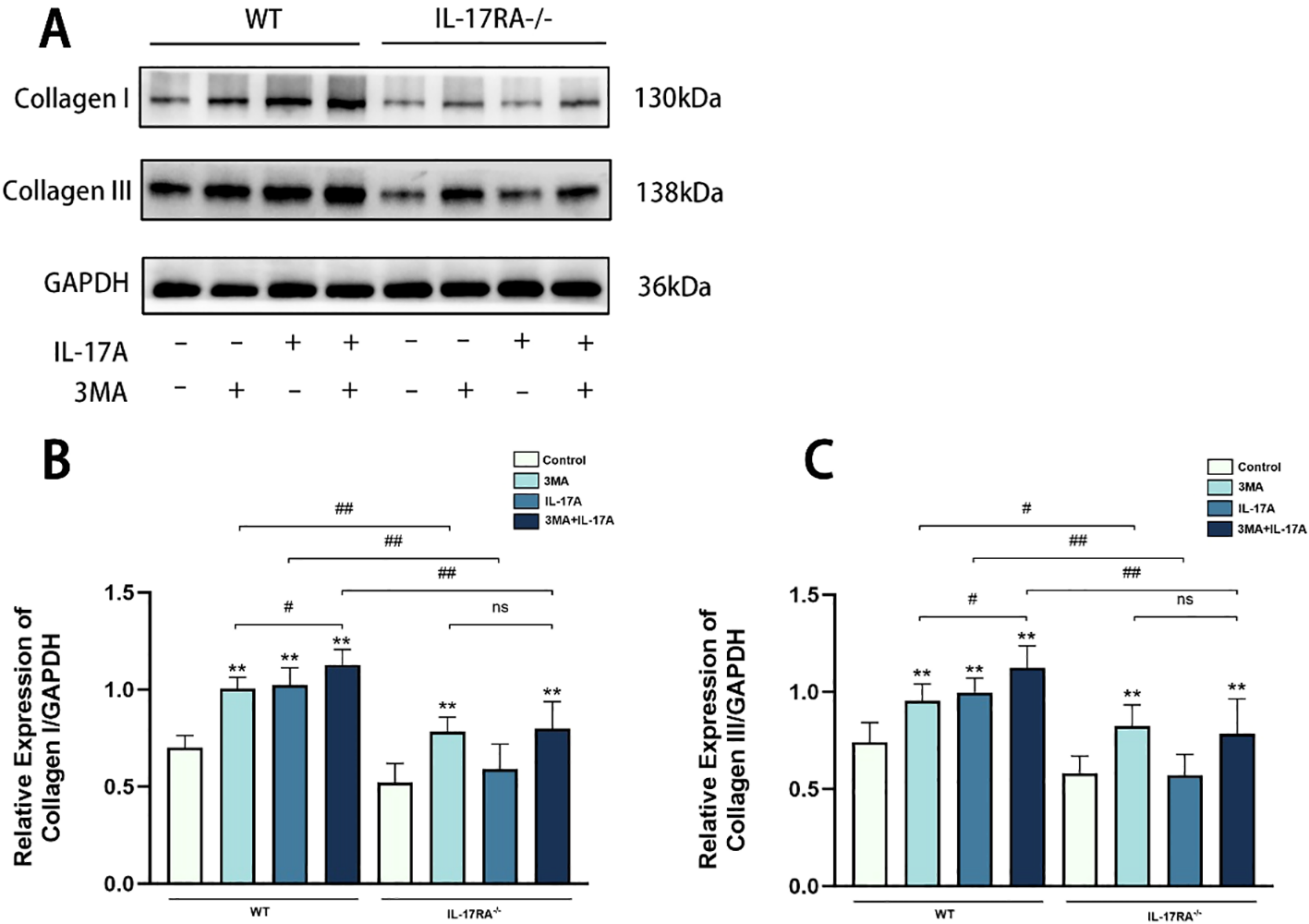
Expression of types I/III collagen in bronchial fibroblasts (x̄ ± SD, n = 5). **(A)** Representative protein bands in the different groups; **(B)** Quantitative determination of collagen I in the different groups; **(C)** Quantitative determination of collagen III in the different groups. Data are presented as mean ± SD. Statistical analysis was performed using two-way ANOVA followed by Tukey’s *post hoc* test. ^**^P < 0.01 versus the genotype-matched Control group; ^#^P < 0.05 and ^##^P < 0.01 between the groups connected by brackets; ns, not significant.

The expression of types I/III collagen proteins in MBFs was examined by WB (**Figures 4A–C**). The following results were obtained: 1) In the WT cells, the protein expression of types I/III collagen was significantly increased in the 3MA, IL-17A, and IL-17A+3MA groups compared with the Control group (P < 0.01). Types I/III collagen proteins showed significantly greater expression in the 3MA+IL-17A group compared with the 3MA group (P < 0.05). 2) In the IL-17RA^-/-^ cells the expression of type I/III collagen protein was increased (P < 0.01) in the 3MA and 3MA+IL-17A groups, compared with the Control group, while no significant difference was observed in the IL-17A group (P ≥ 0.05). No significant differences in expression were seen between the 3MA and the IL-17A+3MA groups (P ≥ 0.05). 3) IL-17RA^-/-^ cells showed reduced collagen expression in the 3MA, IL-17A, and 3MA+IL-17A groups compared with WT cells (P < 0.05 or P < 0.01). These results suggest that IL-17A promoted the secretion of collagen by bronchial fibroblasts.

### 3.5 IL-17A affected inflammatory and profibrotic mediator secretion by bronchial fibroblasts

**Figure 5:**
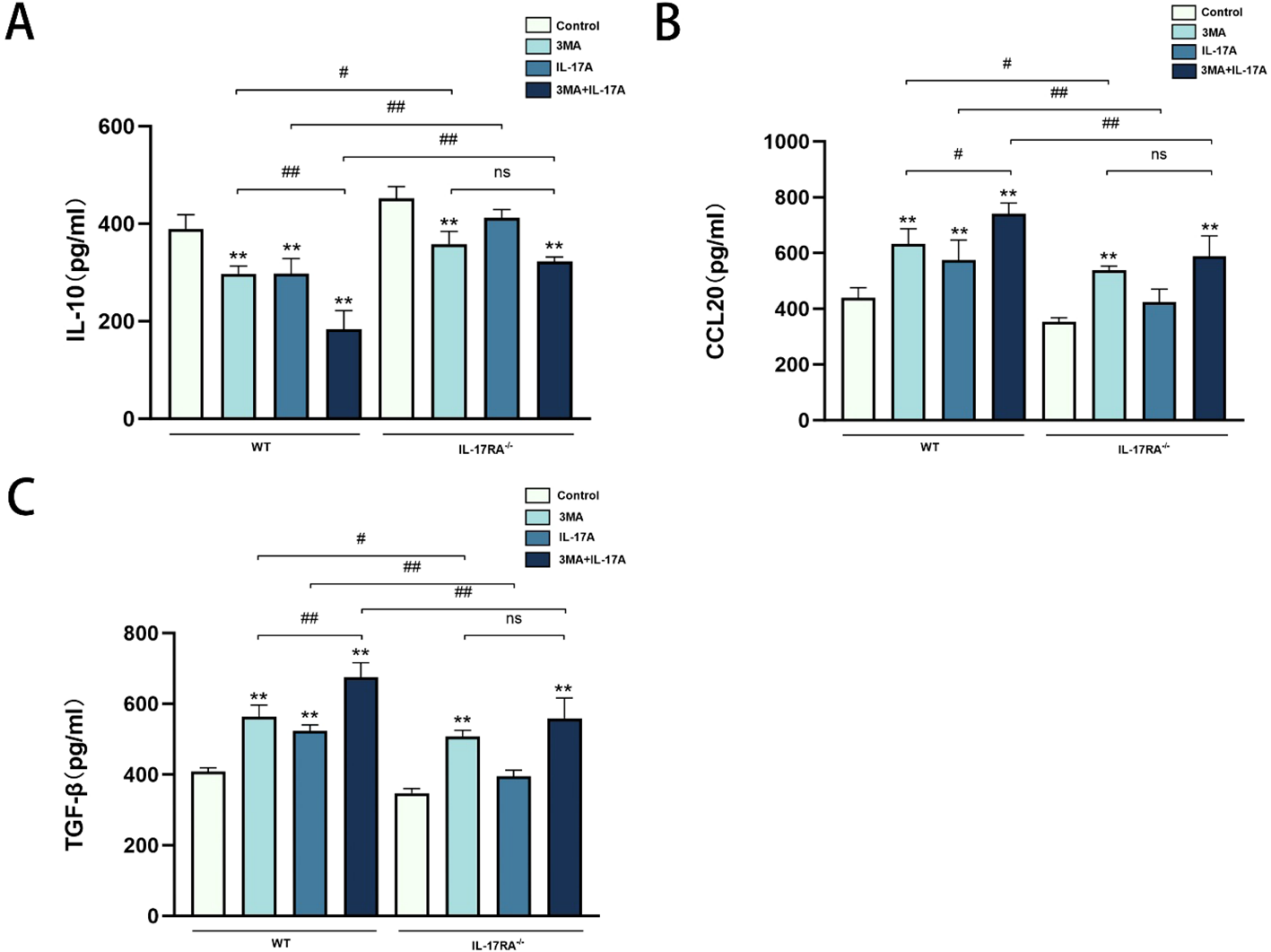
IL-17A regulated inflammatory and profibrotic mediator secretion by bronchial fibroblasts (x̄ ± SD, n = 5). **(A)** Expression of IL-10; **(B)** Expression of CCL20; **(C)** Expression of TGF-β in culture supernatants. Data are presented as mean ± SD. Statistical analysis was performed using two-way ANOVA followed by Tukey’s *post hoc* test. ^**^P < 0.01 versus the genotype-matched Control group; ^#^P < 0.05 and ^##^P < 0.01 between the groups connected by brackets; ns, not significant.

The expression of IL-10, CCL20, and TGF-β in the culture supernatants of MBFs in each group was determined by ELISA (**Figures 5A–C**). The following results were obtained: 1) In WT cells, the expression of CCL20 and TGF-β increased significantly (P < 0.01), while that of IL-10 decreased significantly (P < 0.01) in the 3MA, IL-17A, and 3MA+IL-17A groups compared with the Control group. The expression of CCL20 and TGF-β in the 3MA+IL-17A group increased (P < 0.05 or P < 0.01) while that of IL-10 decreased (P <0.01) compared with the 3MA group. 2) In IL-17RA^-/-^ cells, the 3MA and 3MA+IL-17A groups showed significantly increased CCL20 and TGF-β expression (P < 0.01) and decreased IL-10 expression (P < 0.01) compared with the Control group, while no differences in the expression of these cytokines were observed in the IL-17A group (P ≥ 0.05). No significant differences were observed between the 3MA and 3MA+IL-17A groups (P ≥ 0.05). 3) Compared with WT cells, IL-17RA^-/-^ cells showed significantly reduced CCL20 and TGF-β expression (P < 0.05 or P < 0.01) and increased IL-10 expression (P < 0.05 or P < 0.01) in the 3MA, IL-17A, and 3MA+IL-17A groups. Taken together, these results suggest that IL-17A promoted the inflammatory response in bronchial fibroblasts.

### 3.6 IL-17 promoted the activation of PI3K/AKT/mTOR pathway-related proteins in bronchial fibroblasts

**Figure 6:**
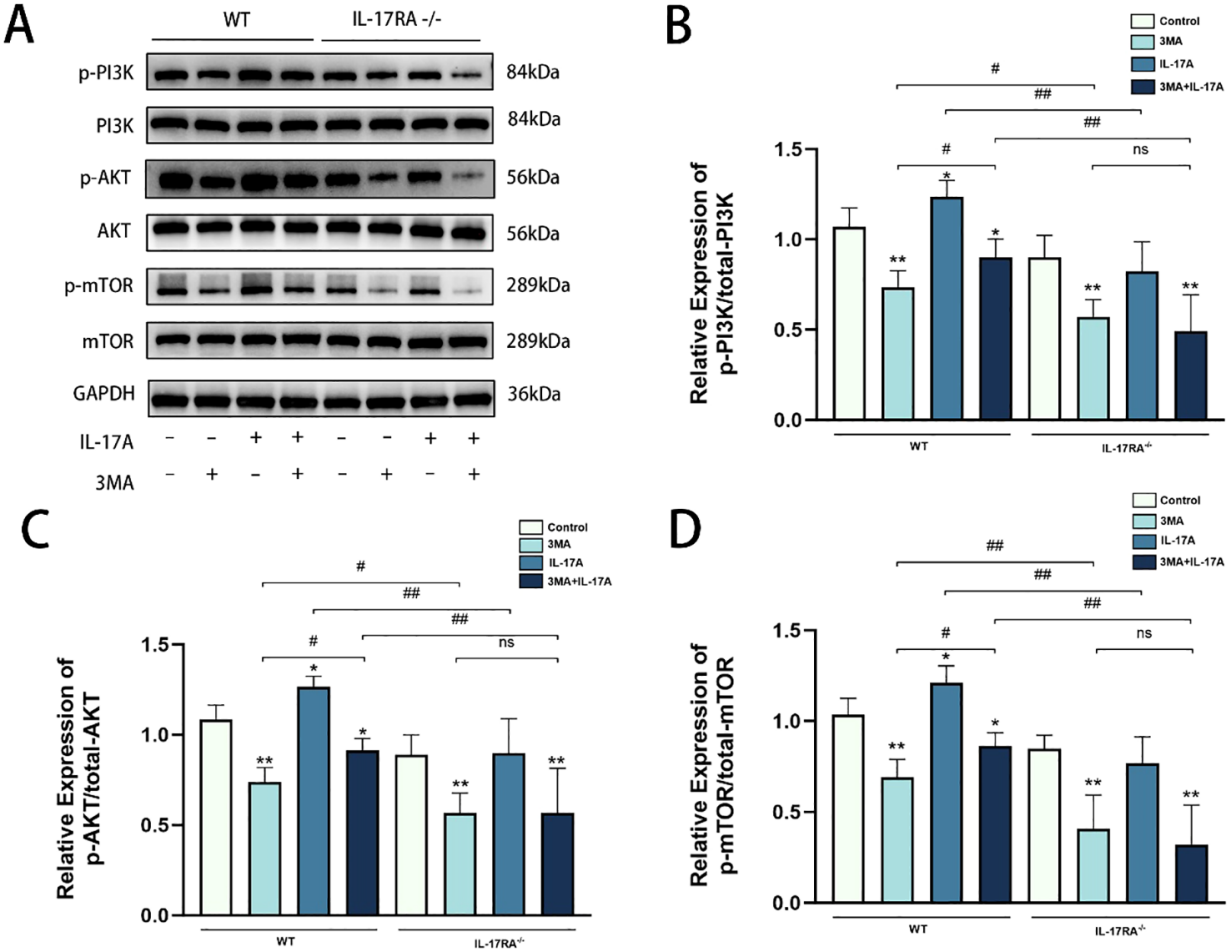
Expression of PI3K/AKT/mTOR pathway-related proteins in bronchial fibroblasts (x̄ ± SD, n = 5). **(A)** Representative protein bands in the different groups; **(B)** Quantitative determination of p-PI3K in the different groups; **(C)** Quantitative determination of p-AKT in the different groups; **(D)** Quantitative determination of p-mTOR in the different groups. Data are presented as mean ± SD. Statistical analysis was performed using two-way ANOVA followed by Tukey’s *post hoc* test. ^*^P < 0.05 and ^**^P < 0.01 versus the genotype-matched Control group; ^#^P < 0.05 and ^##^P < 0.01 between the groups connected by brackets; ns, not significant.

The levels of PI3K/AKT/mTOR pathway-related proteins in MBFs were assessed by WB (**Figure 6**). The following results were obtained: 1) In WT cells, the 3MA and 3MA+IL-17A groups showed significantly decreased phosphorylation of various proteins (P < 0.05 or P < 0.01), while the IL-17A group showed increased phosphorylation of these proteins compared with the Control group (P < 0.05). The 3MA+IL-17A group showed increased phosphorylation of these proteins compared with the 3MA group (P < 0.05), indicating that 3MA reduced activation of the pathway while IL-17A partially reversed this inhibition. 2) In IL-17RA^-/-^ cells, reduced phosphorylation of the proteins was seen in the 3MA and 3MA+IL-17A groups compared with the Control group (P < 0.01), while no significant change was observed in the IL-17A group (P ≥ 0.05) nor were any significant differences seen between the 3MA and 3MA+IL-17A groups (P ≥ 0.05). 3) Compared with WT cells, protein phosphorylation was reduced in IL-17RA^-/-^ cells (P < 0.05 or P < 0.01) in the 3MA, IL-17A, and 3MA+IL-17A groups. This suggests that IL-17A promoted the phosphorylation of PI3K/AKT/mTOR pathway-related proteins in bronchial fibroblasts.

## Discussion

In this study, we constructed a small airway fibrosis model using intratracheal injection of adenovirus-transfected IL-1β in mice, verifying reduction in pulmonary function and pathological changes, confirming a new protocol for studying AR (Zhou et al., 2020). We demonstrated significant collagen deposition around the small airways in AR mice compared to the Control group. IL-17A has previously been shown to promote the secretion of fibronectin, type I collagen, and type III collagen in orbital fibroblasts in thyroid-associated ophthalmopathy (Fang et al., 2016). Consistent with these findings, the present study also demonstrated that IL-17A promoted the secretion of types I and III collagen by bronchial fibroblasts. We showed significantly improved pulmonary function, reduced inflammatory cell infiltration, and less collagen fiber deposition in IL-17RA^-/-^ mice compared to WT mice consistent with reported anti-IL-17 improvement in lung function in COPD mice, which was accompanied by reduced inflammation and collagen deposition around small airways and reduced AR in mouse lung tissue (Fukuzaki et al., 2021). Murine primary bronchial fibroblasts after viral transfection (IL-1b and control) were employed in cellular, mechanistic studies. IL-17A inhibited autophagy in bronchial fibroblasts, similarly to the autophagy inhibitor 3MA, furthermore, combined use of 3MA and IL-17A further increased inhibition. This is in line with studies showing that IL-17A inhibited autophagy in keloid fibroblasts (Park and Heo, 2024). 3MA, like IL-17A, was found to promote the secretion of types I and III collagen by bronchial fibroblasts suggesting that IL-17A may act by autophagy inhibition, to promote the progression of fibrosis.

The present study shares a common research theme with our previous work, namely the involvement of IL-17A-mediated PI3K/AKT/mTOR signaling and autophagy regulation in COPD-related airway remodeling. However, the two studies differ substantially in experimental design, mechanistic focus, and the level of evidence provided. The previous study mainly investigated IL-17RA gain- and loss-of-function in cultured MBFs using lentiviral overexpression or silencing approaches. By contrast, the current study introduced an IL-17RA-deficient mouse model to evaluate the contribution of the IL-17A/IL-17RA axis to airway inflammation, collagen deposition, and lung function impairment *in vivo*. Furthermore, primary bronchial fibroblasts derived from WT and IL-17RA-deficient mice were used to determine whether the effects of exogenous IL-17A on autophagy inhibition, collagen production, inflammatory cytokine secretion, and PI3K/AKT/mTOR activation were receptor-dependent. Therefore, the current study provides genetic and primary-cell-based evidence that complements and extends the previous pharmacological and lentiviral cell-based findings.

Chronic inflammation is also a major characteristic of AR; in asthma IL-10 is reduced (Wu et al., 2024), and Ovalbumin-induced AR is associated with increased expression of TGF-β, IL-13L, and IL-4 in mouse lung tissue (Li et al., 2024). In COPD patients AR is accompanied by massive infiltration of inflammatory cells in bronchial submucosa, airway epithelium, and lung tissue (Mannion et al., 2023), as in our mouse model. In keloid fibroblasts, inhibition of autophagy can promote inflammatory responses (Park and Heo, 2024). We demonstrated in fibroblasts that inhibition of autophagy inhibited the expression of CCL20 TGF-β and IL-10, and, at least in part, this may relate to the mechanism of action of IL-17A.

We showed that IL-17A activated the PI3K/AKT/mTOR pathway to suppress bronchial fibroblast autophagy. Interestingly, 3MA, a PI3K inhibitor, blocked PI3K/AKT/mTOR signaling but ultimately suppressed autophagy, with IL-17A partially reversing the 3MA-induced pathway inhibition. In the present study, 3-MA was used at a concentration of 5 mM. Although 3-MA is generally classified as a PI3K inhibitor, its inhibitory effect on autophagy should be interpreted in the context of the distinct roles of different PI3K classes. Inhibition of Class I PI3K would be expected to suppress AKT-mTOR signaling and thereby favor autophagy induction. However, at 5 mM, 3-MA also potently inhibits Class III PI3K/Vps34, which is required for phosphatidylinositol 3-phosphate generation and autophagosome nucleation. Therefore, under our experimental conditions, 3-MA likely blocked autophagy at the initiation stage by suppressing Class III PI3K/Vps34-dependent autophagosome formation. This interpretation is supported by the classic study by Seglen and Gordon, which showed that 5 mM 3-MA inhibited autophagic/lysosomal protein degradation in isolated rat hepatocytes (Seglen and Gordon, 1982). Moreover, Wu et al. further demonstrated that 3-MA exerts dual and context-dependent effects on autophagy through different temporal patterns of inhibition on Class I and Class III PI3K (Wu et al., 2010). Thus, 3-MA should not be interpreted simply as a general PI3K inhibitor; rather, its effects on autophagy depend on the PI3K class targeted, treatment duration, cellular context, and nutrient status.

**Figure 7:**
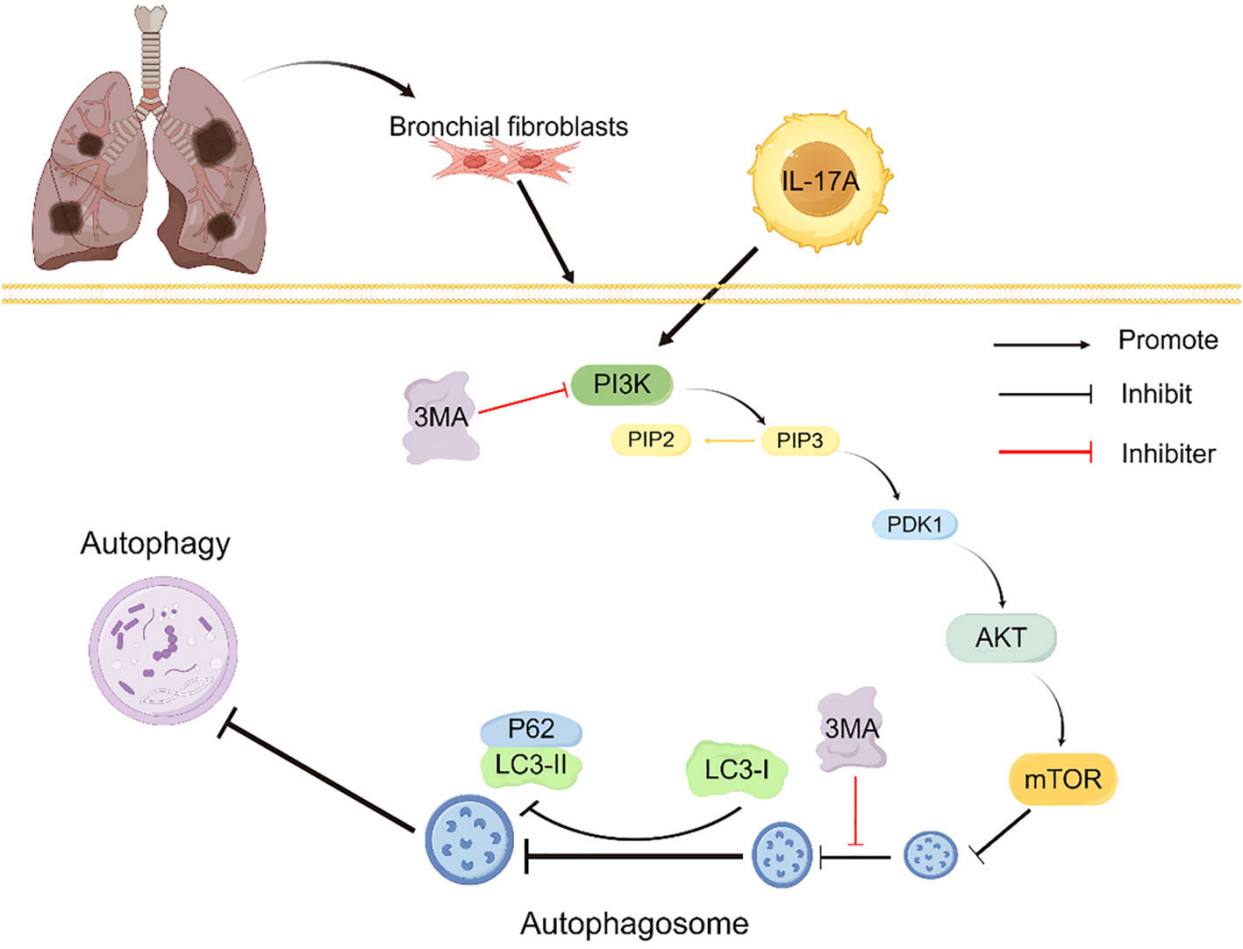
Proposed mechanism of IL-17A-mediated regulation of bronchial fibroblast autophagy in airway remodeling (by Figdraw).

Importantly, 3-MA and IL-17A inhibit autophagy through distinct molecular routes. 3-MA mainly acts as a pharmacological inhibitor of early autophagosome formation through Class III PI3K/Vps34 inhibition. In contrast, IL-17A-mediated autophagy suppression is more likely associated with inflammatory signaling and downstream regulation of autophagy-related pathways rather than direct pharmacological inhibition of Vps34. Thus, although both 3-MA treatment and IL-17A exposure reduce autophagic activity in our study, their upstream mechanisms are different. A schematic summary of the proposed mechanism is shown in **Figure 7**.

Several limitations should be acknowledged. First, although IL-17RA expression was reduced in primary bronchial fibroblasts isolated from IL-17RA^flox/flox^; Sftpc-Cre^+^ mice on a C57BL/6 background, SFTPC-Cre primarily targets SFTPC-positive type II alveolar epithelial cells. Because Cre-reporter lineage tracing, fibroblast-specific Cre detection, and genomic recombination analysis in isolated fibroblasts were not performed, this finding cannot be considered definitive evidence of fibroblast-specific Il17ra deletion and should be interpreted with caution. Nevertheless, the *in vivo* results support the involvement of IL-17RA signaling in airway inflammation and remodeling, whereas the *in vitro* findings indicate that IL-17A regulates autophagy-related responses, collagen expression, inflammatory cytokine secretion, and PI3K/AKT/mTOR pathway activation in primary bronchial fibroblasts.

Second, the precise mechanism by which IL-17A activates the PI3K/AKT/mTOR pathway remains unclear. The present study did not determine whether this regulation occurs through direct molecular interaction with PI3K, IL-17RA-mediated upstream signaling, or other indirect mechanisms. In addition, the *in vitro* experiments were mainly performed using primary mouse bronchial fibroblasts and 3MA as an autophagy inhibitor. Future studies using fibroblast-specific IL-17RA deletion models, additional autophagy modulators or genetic approaches, and human-derived lung structural cells are needed to further validate these findings.

## Conclusion

In conclusion, IL-17A suppressed bronchial fibroblast autophagy and promoted inflammatory and fibrotic responses in association with PI3K/AKT/mTOR pathway activation, thereby contributing to airway remodeling. Targeting IL-17A/IL-17RA signaling or its downstream autophagy-related pathways may provide a potential therapeutic strategy for AR.

## Data Availability

The raw data supporting the conclusions of this article will be made available by the authors, without undue reservation.
